# Overexpression of miR-32 in Chinese hamster ovary cells increases production of Fc-fusion protein

**DOI:** 10.1186/s13568-023-01540-z

**Published:** 2023-05-09

**Authors:** Masoume Bazaz, Ahmad Adeli, Mohammad Azizi, Morteza Karimipoor, Freidoun Mahboudi, Noushin Davoudi

**Affiliations:** 1 Department of Medical Biotechnology, Biotechnology Research Center, Pasteur Institute of Iran, Tehran, Iran; 2 Department of Molecular Medicine, Biotechnology Research Center, Pasteur Institute of Iran, Tehran, Iran

**Keywords:** Fc-fusion, miR-32, microRNA, Productivity, CHO cell engineering

## Abstract

The demand for industrial genetically modified host cells were increased with the growth of the biopharmaceutical market. Numerous studies on improving host cell productivity have shown that altering host cell growth and viability through genetic engineering can increase recombinant protein production. During the last decades, it was demonstrated that overexpression or downregulation of some microRNAs in Chinese Hamster Ovary (CHO) cells as the host cell in biopharmaceutical manufacturing, can improve their productivity. The selection of microRNA targets has been based on their previously identified role in human cancers. MicroRNA-32 (miR-32), which is conserved between humans and hamsters (*Crisetulus griseus*), was shown to play a role in the regulation of cell proliferation and apoptosis in some human cancers. In this study, we investigated the effect of miR-32 overexpression on the productivity of CHO-VEGF-trap cells. Our results indicated that stable overexpression of miR-32 could dramatically increase the productivity of CHO cells by 1.8-fold. It also significantly increases cell viability, batch culture longevity, and cell growth. To achieve these results, following the construction of a single clone producing an Fc-fusion protein, we transfected cells with a pLexJRed-miR-32 plasmid to stably produce the microRNA and evaluate the impact of mir-32 overexpression on cell productivity, growth and viability in compare with scrambled control. Our findings highlight the application of miRNAs as engineering tools and indicated that miR-32 could be a target for engineering CHO cells to increase cell productivity.

**Supplementary Information:**

The online version contains supplementary material available at 10.1186/s13568-023-01540-z.

## Introduction

The biopharmaceuticals market was expanded significantly, and biotherapeutics' demands, especially for recombinant proteins and antibodies, are expected to rise further in the future. In this respect, it is critical to develop more efficient cell lines as host for recombinant protein production (Szkodny and Lee 2022; Fischer et al. 2015b; Mullard 2021). Chinese Hamster Ovary (CHO) is a widely used mammalian host cell for the production of complex, glycosylated, and hard to express therapeutics (Keysberg et al. 2021). Over recent decades, various genetic manipulating approaches have been applied to make significant improvements in CHO cell productivity. Because of the strong relationship between specific productivity and host cell proliferation, viability, and culture duration, a variety of genetic engineering approaches have been used to optimize these characteristics through beneficial genes or genomic knock-out of undesirable genes. (Fischer et al. 2015a; O’Flaherty et al. 2020; Keysberg et al. 2021; Kumar et al. 2007).

MicroRNAs are a group of noncoding RNAs that negatively regulate gene expression by targeting the 3′ untranslated region (3’UTR) of mRNAs and prevent translation. They have long been the focus of research on gene therapy and cancer (Huhn et al. 2019; Romano et al. 2017; Maccani et al. 2014). Several studies indicate that deregulation of microRNAs in CHO cells can increase cell productivity (see Table 1) by interfering with a variety of cellular mechanisms such as proliferation, post-translational modification, secretion, and apoptosis. (Singh et al. 2022; Bazaz et al. 2022; Koh et al. 2009).

**Table 1:** Previous used microRNAs for modifying CHO cells

miR	Product	Effect or Target	References
miR-23a miR-377	Lysosomal sulfatase	Specific Activity	Amadi et al. 2020
miR-106b	IgG	Inhibition of CYLD	Xu et al. 2019
miR-744	mAb	Productivity	Raab et al. 2019
miR-143	mAb/SEPA	Productivity	Schoellhorn et al. 2017
miR-92a	anti-HER2 IgG	Protein secretion	Loh et al. 2017
miR-483	mAb	Productivity	Emmerling et al. 2016
miR-30	SEAP	Ubiquitin pathway	Fischer et al. 2015a, 2015b
miR-23	SEPA	metabolism	Kelly et al. 2015b
miR-34a	SEPA	Cell Growth	Kelly et al. 2015a

One of the screening methods to select a microRNAs which could potentially increase cell productivity is using previously discovered microRNAs in human cancer research that affect cell growth properties. (Inwood et al. 2018; Stiefel et al. 2016; Lim et al. 2006; Peng and Croce 2016) Kelly and colleagues, reported the effect of miR-23 depletion on the productivity of SEPA-expressing CHO cells based on a prior understanding of miR-23's pro-apoptotic and anti-proliferative capabilities (Kelly et al. 2015a, 2015b).

MicroRNA-32 (miR-32) is one of the important regulators in tumorigenesis, and its oncogenic effects have been reported in different cancer studies. It was demonstrated that miR-32 affects and regulates cell growth by targeting various mRNAs involve in cell proliferation (Zhang et al. 2020; Zhang et al. 2019; Xia et al. 2017; Xia et al. 2015; Li and Wu 2016). In this study, we evaluated the effect of stable overexpression of miR-32 on growth, viability, and productivity of the CHO-k1 cell, which stably expresses an Fc-Fusion recombinant protein (VEGF-trap). VEGF-trap (Aflibercept) is an anti-VEGF drug. This homomeric fusion protein is expressed in CHO cells and is made up of the constant region of human IgG1, the third Ig domain of human VEGFR2, and the second Ig domain of human VEGFR1. (Keshet et al. 2021; Holash et.al. 2022). The molecular weight of this molecule was reported about 115–150 KD (Sivertsen et al. 2018; Khalili et al. 2016).

## Material and methods

### Expression vector construction

To make the Fc-fusion expression constructs, the pCR2.1 plasmid containing *VEGF-trap* gene sequence was digested by *Kpn*I and *Not*I and the gel extracted DNA was subcloned into the *Kpn*I and *Not*I sites of the pTracer-CMV2 plasmid DNA (Invitrogen, USA). The data not shown. The plasmid also contains coding sequence for zeocin resistance protein as selectable marker and green fluorescent protein (GFP). The obtained construct was used as a plasmid DNA for transfection of  CHO-K1 cells.

### Development of a stable cell line producing VEGF-trap

The adherent CHO-K1 cells were cultured in Dulbecco's Modified Eagle Medium-F12 (DMEM-F12, Gibco) supplemented with 10% Fetal Bovine Serum (FBS, Gibco) at 37 °C, 5% CO2, and 85% humidity. Every 3 or 4 days, the cells were passaged. In order to create stable CHO-K1 cell line that express the VEGF-trap (an Fc-fusion protein), 8 × 10^4^ cells were seeded in 24-well cell culture plated 24 h before transfection. The cells transfected with and 0.5 μg of pTracer-CMV2 vector include VEGF-Trap Gene using lipofectamine 3000 (Invitrogen, USA) according to the manufacturer’s protocols. Prior to transfection, all plasmid DNAs were linearized using *Sca*I restriction enzyme. Two days after transfection, the cells were transferred in 6-well plate and subjected to selection pressure for 2 weeks in a medium containing 200 µg/ml Zeocin (Invitrogen, USA). The Culture medium contains antibiotic, exchanged every 3-days. Fluorescent microscope (OPTICA) and Fc reagent ELISA kit (EK000095-20310, Syd Labs, USA) was used for analysis of generated cell pool and identifying the expression of GFP and an Fc-fusion respectively. Clonal selection performed based on single limiting dilution of cell pool. To perform limiting dilution step, the cells detached and diluted step by step to obtain on cell 10 cell/ml and each 0.1 ml of this suspension transferred into one well of 96-well cell culture plate. The wells monitor about then days. The wells containing two or more clones were excluded and the remained single clones have been screened based on their Fc-fusion expression assay using ELISA and population homogeneity assay using flowcytometry system.

### VEGF-trap purification and SDS-PAGE

To purify the Fc-fusion protein, 3 × 10^6^ cells were cultured in a T-75 cell culture flask. The supernatant culture media was harvested on day 6 of batch cultivation. VEGF-ELISA applied to ensure the binding of VEGF to expressed Fc-fusion. As positive and negative controls, respectively, we used CHO-K1 supernatant and the standard VEGF-trap protein Eylea (Bayer, Germany).

To purify the VEGF-trap molecule, affinity chromatography based on protein A binding to Fc region of recombinant protein was used. A 1 ml purification column, HiTrap Protein A HP (GE healthcare) was utilized to purify the Fc-fusion from harvested supernatant based on affinity binding of protein A to Fc region of recombinant protein according to manufacturer instructions. Briefly, after connecting the column to the FPLC System (BioLogic DuoFlow system, Bio-Rad) and washing it with 10 ml of binding buffer (20 mM sodium phosphate), the sample were pumped onto the column. We used 10 ml of binding buffer to wash the column after loading the sample. Subsequently, Fc-Fusion protein were eluted using elution buffer (0.1 M citric acid, pH 3–6) and collected in 1.5 microtubes containing 150 µl of neutralizing buffer (1 M Tris–HCl, pH 9.0) per 1 ml of fraction. All the buffers and media filtered through a 0.45 µm filter before use, and the flow rate was kept at 1 ml/min during the whole purification process. A fraction of collected samples subjected to 12% SDS-PAGE to analyze the purity and size of VEGF-trap in compare with standard protein, Eylea (Bayer, Germany).

### Enzyme linked immunosorbent assay

The Human Fc ELISA Reagent kit (EK000095-20310, sydlabs, USA) was used to measure the concentration of Fc-fusion. To perform ELISA, 96-well ELISA plates (44-2404-2, Nunc MaxiSorpTM flat-bottom) were coated with the capture antibody (anti-human Fc fragment) diluted in coating buffer (PBS, pH 7) and incubated at 4 °C overnight. The plates washed one time with PBS (phosphate buffer saline) and then blocked for an hour at room temperature (RT) with blocking buffer (PBS supplemented with 3% BSA) (A2153, Sigma-Aldrich, USA). Following a PBS wash, samples and diluted standards were added. The plate was then incubated for an hour at RT. After washing with washing buffer (PBS with BSA and Tween), HRP conjugated antibody was added to each well at a 1:1000 dilution in diluent buffer (PBS supplemented with 1% BSA). After 1-h incubation at RT and washing, TMB solution was added. The enzymatic process was stopped with 3 m HCL, and plates were analyzed at 450 nm using an Epoch Microplate Spectrophotometer (Biotek).

In order to make sure that the expressed Fc-fusion protein binds to VEGF, we performed a VEGF-ELISA as described above but coated with a VEGF1 protein (Biosera, France) at a concentration of 2 μg/ml.

### MicroRNA selection and plasmid construction

In order to choose a microRNA as a candidate for CHO cell engineering, all the upregulated microRNAs in human cancer have been collected from the KEGG database (MicroRNAs in Cancer, www.genome.jp/pathway/hsa05206). and screened based on their previously described roles as cell proliferation regulators in cancer research. which have conserved sequence in humans and hamsters (*Crisetulus griseus*). The selected microRNA(mir-32) obtained by using the MiRBase (www.mirbase.org) and analyzed also by miRWalk, DIANA Tarbase, miRDB, and miRmap.

The sequence of *mir-32*, was inserted into the pLexJRed vector (Open Biosystems, USA) between the *Mlu*l and *Xho*l sites following PCR amplification of extracted genomic DNA using the forward and reverse primers 5’-CTACGCGTGAGAATCGATGGCATACAC-3’ and 5’-TACTCGAGGCATGACCAGACAGTGATAGTG-3’, respectively (Additional file 1: Fig. S2). The sequence of clones was confirmed using the standard DNA sequencing methods.

### Development of stable CHO cell line overexpressing miR-32

In order to stably overexpress miR-32 in CHO-VEGF-trap cells, the pLexJRed-mir-32 vector was transfected into the cells. A control plasmid phum-Red-GFP-Scrambled (HumDiagnostics, Iran), pLexJRed-containing scrambled microRNA, was also transfected into another group of cells, according to the protocol previously described. Briefly, 8 × 10^4^ CHO-VEGF-trap cells were seeded in 24 well-cell culture plates. One group of cells was transfected with 0.5 μg pLexJRed-miR-32 plasmid, and the other was transfected with 0.5 μg pLexJRed plasmid DNA containing scrambled microRNA using Lipofectamine3000 (Invitrogen, USA)0.48 h after transfection, the cells were transferred into 6-well plates and cultivated in selection media containing puromycin (3 μg/ml) for 2 weeks to generate stable cell pools. Fluorescence detection and quantitative real-time PCR were used to confirm the success of transfection and the expression of miR-32 in cell pool, respectively. Non-transfected CHO-VEGF-trap cells were used as an additional control for the comparative examination of productivity, growth, and viability in order to enhance the accuracy of the results.

### Analysis of cell growth and productivity

To investigate the productivity, growth, and viability of CHO-VEGF-trap-miR-32 (miR-32) and controls (scrambled transfected (Sc) and non-transfected CHO-VEGF-trap (NT), the cells were grown in batch culture conditions for 10 days for each group, cells were seeded at 30% confluency into 12-well plates. Total cell counts were determined using a hemacytometer every day. The trypan-blue dye exclusion method was used to determine viable cell density and cell viability. Cells from each group were seeded into a six-well plate at 30% confluence and cultivated for 6 days for specific productivity (Qp) analysis. The number of cells and Fc-fusion titer were assessed on the first and last days for each group. The specific productivity (fg/cell/day) was estimated using the following equation, where P stands for the Fc-fusion titer, X for the number of cells, and t for the time of culture in a day (Mohammadian et al. 2019).

$$Qp=\frac{P2-P1}{\frac{(X2+X1)}{2}\times (t2-t1)}$$

### RNA extraction and quantitative Real-Time-PCR

Total RNA was extracted from 3 groups of cells, miR-32, NT, and Sc, using the RiboEx (GeneAll, Korea) according to the manufacturer's protocol. Briefly, the pellets of 2.5 × 10^6^ cells were mixed in 1 ml RiboEx, and 0.2 ml of chloroform was added to each tube. The aqueous phase was transferred to a new tube after centrifuging the samples at 12,000 × *g* for 15 min at 4 °C. Following isopropanol treatment and ethanol precipitation of RNAs, the RNA pellets were air-dried then dissolved in RNase-free water. The quantity and quality of RNA was measured using a nanodrop at OD 260/280 nm. 500 ng of total RNA were reverse transcribed into cDNA by the FIREScript RT cDNA Synthesis Kit (Solis BioDyne, Estonia). Specifically designed stem loops were used for the reverse transcription of microRNAs. Table 2 shows the sequences of primers and stem loops.

**Table 2:** Sequences of Stem loops and primers

Name of the gene	Sequence of oligonucleotides from (5’–3')
miR-32-5p Stem loop	GTCGTATCCAGTGCAGGGTCCGAGGTATTCGCACTGGATACGACGCAACT
miR-32-5p Forward	CGTCCGTATTCCACATTACTAAGT
U6 Stem loop	GTCGTATCCAGTGCAGGGTCCGAGGTATTCGCACTGGATACGACAAAATATGG
U6 Forward	GCAAGGATGACACGCAAATTC
Universal Reverse	GTGCAGGGTCCGAGGT

In order to perform Q-PCR, mir-32-5p forward and universal reverse primers were employed together with a sybergreen master mix (Ampliqon). The U6 gene was used as internal reference gene. The reactions were conducted in StepOnePlus real-time PCR System (Applied Biosystems, USA) The following thermal program was used: initial denaturation at 95 °C for 15 min, and 40 cycles of denaturation at 95 °C for 15 s, and annealing/elongation at 60 °C for 60 s. The fold change (FC) of miR-32 expression level between controls and treatment groups was calculated using the delta-delta Ct method (FC = 2^-(ΔΔCt)^). The amplification efficiency was determined by applying linear regression analysis using LinRegPCR program (Untergasser et al. 2021).

### Statistical data analysis

All the comparative experiments were performed in triplicate. Statistical analysis was carried out using Excel and GraphPad PRISM version 9. In order to compare group differences and calculate the p-value between conditions, One-Way analysis of variance (ANOVA) with a significance level of 0.05 was utilized.

## Results

### Development of stable single clones producing Fc-fusion

**Fig. 1:**
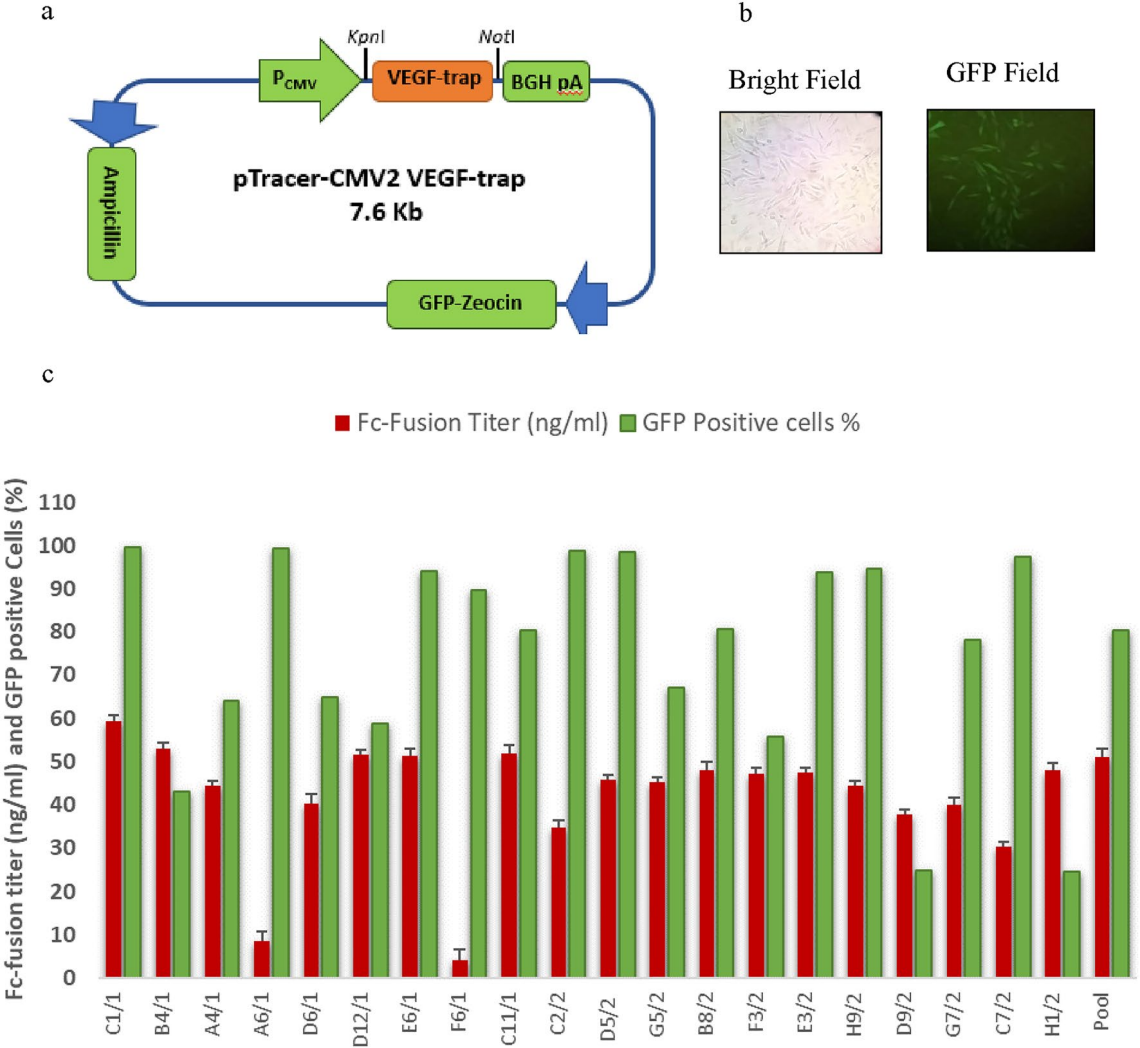
Expression of FC-fusion in CHO-K1 cells and clonal selection results. **a** Schematic view of the pTracer-CMV2-VEGF-trap construct. **b** Indicates the result of fluorescent imaging of pTracerCMV2-Aflibercept transfected cells after antibiotic selection of cell pool. **c** Indicates the derivative single clones after limiting dilution by their Fc-fusion expression and population heterogeneity based on percentage of GFP positive population. More details of flow cytometry results are shown in Additional file 1: Fig. S1. Error bars represent standard deviation of technical replicate (n = 3) of ELISA test

The CHO-K1 cells that had been transfected with the pTracer CMV2-Fc-Fusion gene (Fig. 1a) were selected using antibiotic pressure (Zeocin). Successfully transfected cells appeared green under the GFP field because the vector contains the GFP sequence as a marker gene (Fig. 1b). Antibiotic selection of transfected cells using Zeocin for 3 weeks resulted in the generation of a stable cell pool. Following limiting dilution, the single clones were examined for recombinant protein expression and population heterogeneity. Figure 1c shows the outcomes of Fc-fusion expression and the percentage of GFP-positive cells obtained for each clone. The C1/1 single clone that had the highest expression of Fc-fusion and the most population homogeneity was selected for further experiments (microRNA transfection).

### The secreted Fc-fusion bound to the VEGF

**Fig. 2:**
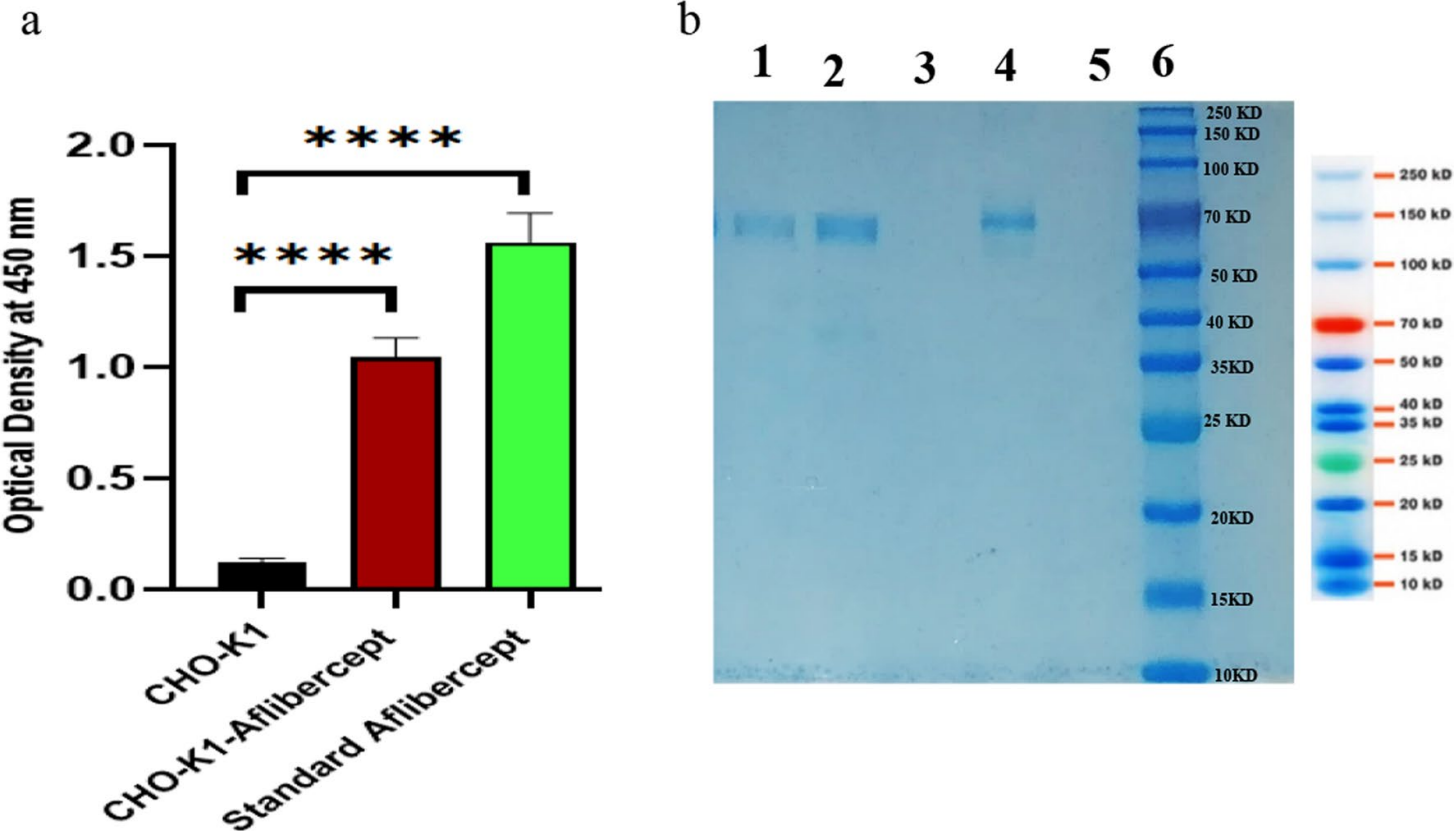
VEGF-ELISA and SDS-PAGE results. **a** Indicates the result of VEGF-ELISA. The standard aflibercept and CHO-K1 supernatant have been used as positive and negative control respectively. Error bars represent standard deviation of technical replicate (n = 3). The stars denote the significance of differences (p < 0.05). **b** Indicates the reduced SDS-PAGE gel result of purified VEGF-trap produced by selected single clone. The samples were subjected to reducing (R) SDS-PAGE assay and the band of approximately 70 kDa was observed, as expected, L1 and L2 are different purified fractions, L4 standard aflibercept, L6, protein marker (10–250 kDa) L3 and L5 are empty

Additional analysis of Fc-fusion produced by the C1/1 clone was done using SDS-PAGE and VEGF-ELISA. The VEGF-ELISA result indicated that the secreted Fc-fusions from the CHO-K1 clone C1/1 could bind to VEGF protein coated on the ELISA plate at a concentration of 2 μg/ml. The CHO-K1 supernatant was used as a negative control. (Fig. 2a). The result of reduced SDS-PAGE of purified VEGF-traps also confirmed the size of our protein relative to standard protein. As shown in Fig. 2b, the size of both the expressed VEGF-trap as well as the standard Eylea is around 70 KD. VEGF-Trap is a homodimeric glycoprotein and the disulfide bonds are responsible to keep it in its dimeric form; Due to using reduced SDS-PAGE acryl amid gel, we can see the protein in monomeric form on the stained gel.

### MicroRNA expression was increased in cells transfected with miR-32

**Fig. 3:**
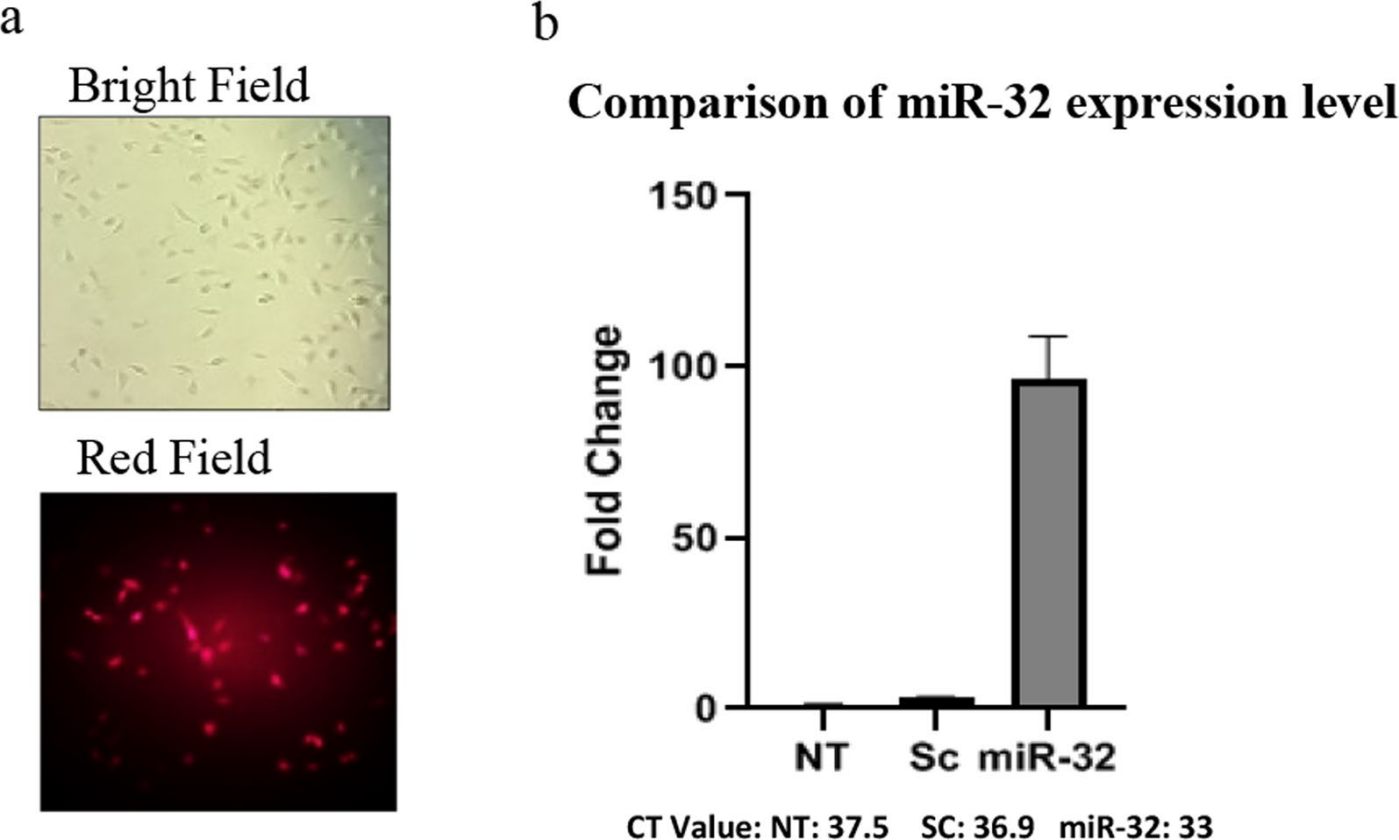
Overexpression of miR-32 in CHO-VEGF-trap Cells. **a** Indicates fluorescent microscopy of miR-32 transfected Cells. **b** Indicates the result of comparative analysis of miR-32 expression level using qRT-PCR in miR-32 transfected Cells in compare with non-transfected (NT) and scrambled transfected (Sc) cells, the miRNA expression is illustrated as fold-change relative to the controls at day 6 of batch cultivation and normalized to U6 snoRNA. Error bars represent standard deviation of technical replicate (n = 3). The significance of differences between miR-32 transfected cells and controls was verified (p < 0.05)

Fluorescence microscopy provided evidence of transfection. The miR-32 transfected CHO-VEGF-trap cells appeared red under a fluorescent lens (Fig. 3a). After puromycine selection of transfected cell and making stable cell pools, the qPCR findings showed that miR-32 expression level was significantly higher in mir-32 transfected cells than scrambled microRNA transfected cells and non transfected cells. Statistical analysis of qPCR results following 6 days batch cultivation of cells and microRNA extraction, indicated that, the miR-32 level in CHO-Fc-fusion-miR-32 cells, increased about 90-fold compared with scrambled control (Sc) and CHO-Fc-fusion cells as an additional control (NT) (Fig. 3b).

### Stable overexpression of miR-32 enhanced the specific productivity

**Fig. 4:**
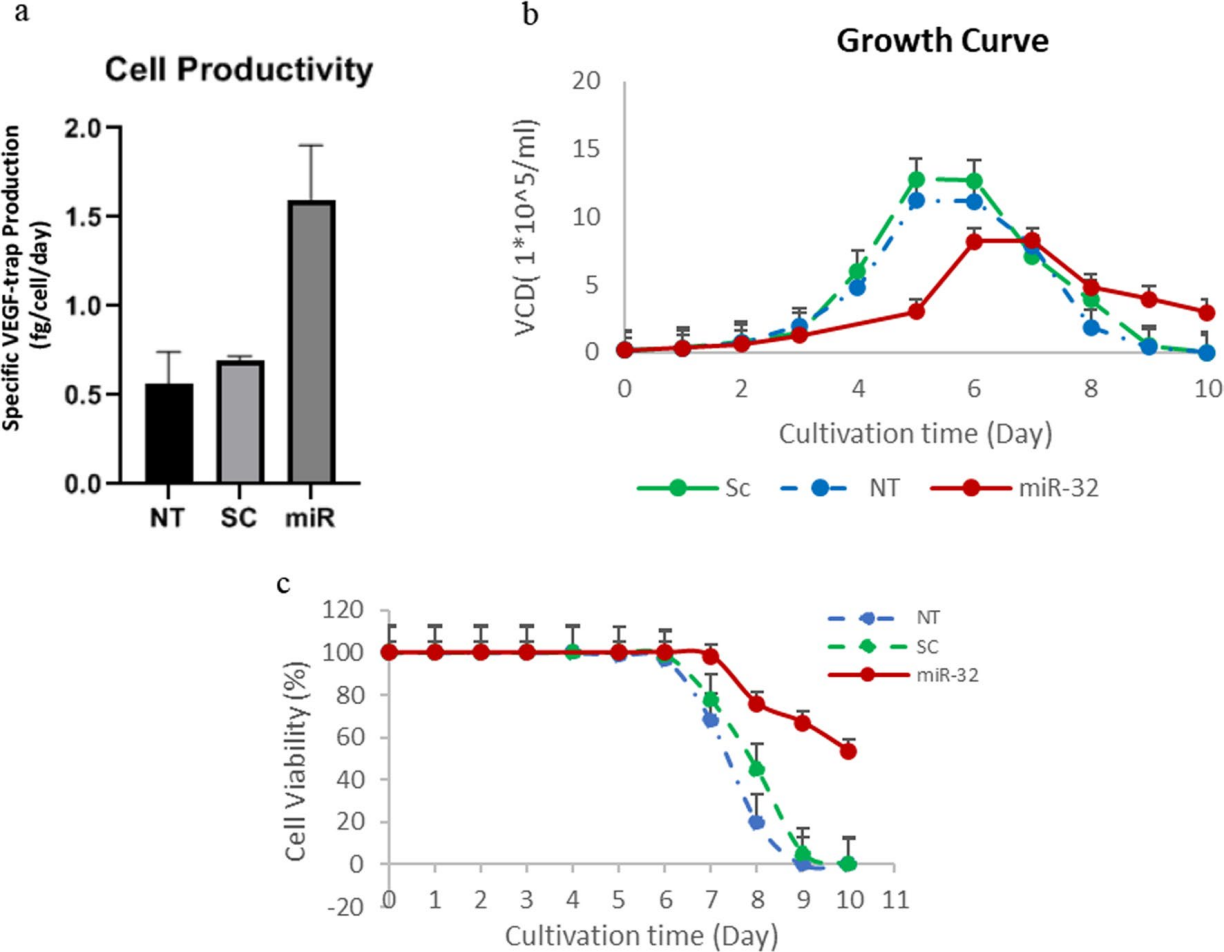
Comparative analysis of productivity, cell growth and viability. **a** A comparative analysis of productivity of mir-32 transfected CHO cells with NT and Sc controls reveals 1.8-fold increase in productivity of miR-32 overexpressed cells. Error bars represent standard deviation of technical replicate (n = 3). The significance of differences between the experimental and control groups was verified (p < 0.05). **b**, **c** The effect of stable overexpression of miR-32 on growth rate and viability of VEGF-trap expressing CHO cells during 10-days batch culture, respectively. Each point in b and c represents viable cell density and cell viability of an individual day, respectively

The growth profile, viable cell density (VCD), and Fc-fusion titer of miR-32 overexpressed cells were compared with controls, NT, and Sc during 10 days of batch cell culture in order to determine the impact of miR-32 overexpression on cell productivity and viability. As shown in Fig. 4a, the specific productivity of miR-32 overexpressed cells was 1.8 times higher than controls. All the experiments were performed in triplicate and statistical analysis indicated that productivity was increased significantly in CHO-VEGF-trap-miR-32 cells in comparison to controls (p value < 0.05).

### Mir-32 overexpressed CHO cells had higher cell viability

Beside the productivity, we compared the growth behavior and batch culture longevity of each cell group. As shown in Fig. 4b, the growth profile of miR-32-overexpressed CHO-Fc-fusion cells was not similar to Sc and NT controls. It also reveals that, the viability of miR-32 overexpressed cells is greater than 65% until the day 9 of batch culture. In contrast, the viability of NT and Sc controls drops to less than 65% two days after the stationary phase (on day 7 of batch culture). A comparison of the log phase and batch culture durations of each cell group revealed that miR-32 transfected cells had increased log phase and batch culture longevity also.

## Discussion

Mammalian cell lines, especially Chinese Hamster Ovaries, are frequently employed to produce complex, glycosylated, and hard-to-express recombinant therapeutics (Keysberg et al. 2021). Numerous genetic manipulation strategies have been used to improve the productivity of these cells. Over the last decade, microRNAs have received a lot of attention as a tool for CHO cell engineering and productivity enhancement through altering different cell mechanisms such as growth, post-translational modification, apoptosis, and cell proliferation (Inwood et al. 2018; Jadhav et al 2013). Previously, we reviewed recent developments in miRNA engineering in CHO cells. (Bazaz et al. 2022). In this study, we selected a known upregulated microRNA in cancer as a novel candidate to improve the productivity of CHO cells. As a result, we successfully constructed a CHO single clone that produces an Fc-fusion protein (VEGF-trap) and showed that miR-32 overexpression in the CHO-VEGF-trap cells affects the viability, growth and specific productivity.

There are many studies related to productivity improvement through microRNA engineering of CHO cells. We consider miR-32 as a novel target for CHO cell engineering which is an important regulator in tumorigenesis (Yan et al. 2015; Xia et al. 2017), and its effect on cell growth and proliferation was reported in different human cancers. In 2019, an investigation on cervical cancer and microRNA revealed that miR-32-5p can regulate cell proliferation by targeting HOXB8, homeobox B8, which encodes a nuclear protein with a homeobox DNA-binding domain (Liu et al. 2019). Zhang and colleagues suggested that miR-32 overexpression inhibits ovarian cancer cell proliferation, by targeting B and T lymphocyte attenuator (BTLA), an activator of phosphatidylinositol-3 kinases (PI3K) which mediates cell proliferation and survival (Ning et al. 2021; Zhang et al. 2020). Zeng et al. reported that by targeting SMG1, a tumor suppressor gene, miR-32 promotes ovarian cancer cell proliferation (Zeng et al. 2020).

In evaluation of studies and researches over the past decade it was discovered that miR-32 plays a wide range of regulatory roles in biological events, especially in tumorigenesis by targeting proteins such as E2F transcription factor 5, phosphatase and tensin homolog (PTEN), F-Box and WD repeat domain containing 7 (FBXW7), SRY-Box Transcription Factor 9 (Sox9), and Twist (Xu et al. 2019; Yan et al. 2015; Liu et al. 2021; Yang et al. 2019; Xia et al. 2017).Based on the regulatory role of miR-32 in human cancer and the conserved sequence of miR-32 between humans and hamsters (*Crisetulus griseus)* as well as its effects on cell proliferation, growth, and viability we hypothesized that overexpression of miR-32 in our CHO-VEGF-trap cells may increase the cell viability and the recombinant protein yield.

Our results also indicated that the specific productivity of miR-32 overexpressed cells have increased significantly1.8-fold more than controls. The qPCR results also showed that miR-32 transfected cells had a 90-fold increase in microRNA expression. The cell growth graph also revealed that cell survival and batch culture time of miR-32 overexpressed cells increased in comparison to control. Previous research on CHO cell productivity also found that controlling cell proliferation, which causes batch culture longevity, can increase the yield of recombinant protein (Kumar et.al. 2007). Based on the significantly increased level of miR-32 in our cells and the regulatory role of miR-32 in cell proliferation, it appears that increased specific productivity of CHO cells is due to miR-32 overexpression. Several attempts to improve the productivity of hard-to-express proteins in CHO cells successfully increased the yield through microRNA overexpression. XU and colleagues indicated that overexpression of miR-106b in CHO cells expressing IgG achieved about a 0.66-fold increase in product titer (Xu et al. 2019). Schoellhorn et al. which investigated the effect of stable overexpression of miR-142 on the productivity of SEPA and IgG-producing CHO cells, reported the 70-fold increase in miR-142 level could cause about a 1.3-fold enhancement in the volumetric productivity of CHO cells (Schoellhorn et al. 2017).

In conclusion, this is the first report describing the effect of miR-32 overexpression on CHO cell productivity. The current study indicated that the genetic manipulation of VEGF-trap producing CHO cells with stable overexpression of miR-32 successfully enhanced the specific productivity of cells and also demonstrated that miR-32 could be a candidate for CHO cell engineering and improving the productivity of hard-to-express proteins. Our data suggested that other microRNAs with the regulatory role in growth and productivity could be candidates for CHO cell engineering and productivity improvement and also in other industrial host cells such as HEK, SP2/0.

## Supplementary Information

Additional file 1: Fig. S1. The flow cytometry results of CHO-Afli single clone screening based on percentage of GFP positive population. Following limiting dilution of cell pool. The single clones detached and analyzed using flow cytometry system. Fig. S2. Schematic picture of constructed mir-32 expression plasmid. The purified PCR product of mir-32 gene was double digested with two different restriction enzymes and was inserted into the pLexJRed vector between the Mlul and Xhol sites.

## Data Availability

Raw data supporting the findings of this study are available upon reasonable request from the corresponding author.

## Abbreviations

CHO: Chinese hamster ovary
miR-32: MicroRNA-32
SnRNAs: Small non-coding RNAs
3’UTR: 3′ Untranslated region
FBS: Fetal Bovine Serum
GFP: Green fluorescent Protein
HRP: Horse Radish Peroxidase
VCD: Viable cell density
BTLA: B and T lymphocyte attenuator
PI3K: Phosphatidylinositol-3 kinases
PTEN: Phosphatase and tensin homolog
FBXW7: F-Box and WD repeat domain containing 7
Sox9: SRY-Box transcription factor 9
