# Supplementary material for: Overexpression of miR-32 in Chinese hamster ovary cells increases production of Fc-fusion protein
Source: AMB Express. 2023 May 9;13:45. doi: 10.1186/s13568-023-01540-z (PMC10170017; doi:10.1186/s13568-023-01540-z)
Supplement: Supplementary file 1 — Additional file 1: Fig. S1. The flow cytometry results of CHO-Afli single clone screening based on percentage of GFP positive population. Following limiting dilution of cell pool. The single clones detached and analyzed using flow cytometry system. Fig. S2. Schematic picture of constructed mir-32 expression plasmid. The purified PCR product of mir-32 gene was double digested with two different restriction enzymes and was inserted into the pLexJRed vector between the Mlul and Xhol sites. [file 13568_2023_1540_MOESM1_ESM.pdf]

Overexpression of MiR-32 in Chinese Hamster Ovary Cells Increases Production of Fc-fusion Protein

Masoume Bazaz[1], Ahmad Adeli, Mohammad Azizi[1], Morteza Karimipoor[1], Freidoun Mahboudi[1], Noushin Davoudi[1]

[1] Department of Medical Biotechnology, Biotechnology Research Center, Pasteur Institute of Iran, Tehran, Iran

Corresponding Author: Noushin Davoudi

Email address: davoudi@pasteur.ac.ir

Running Title: Evaluation the effect of miR-32 on CHO cell productivity

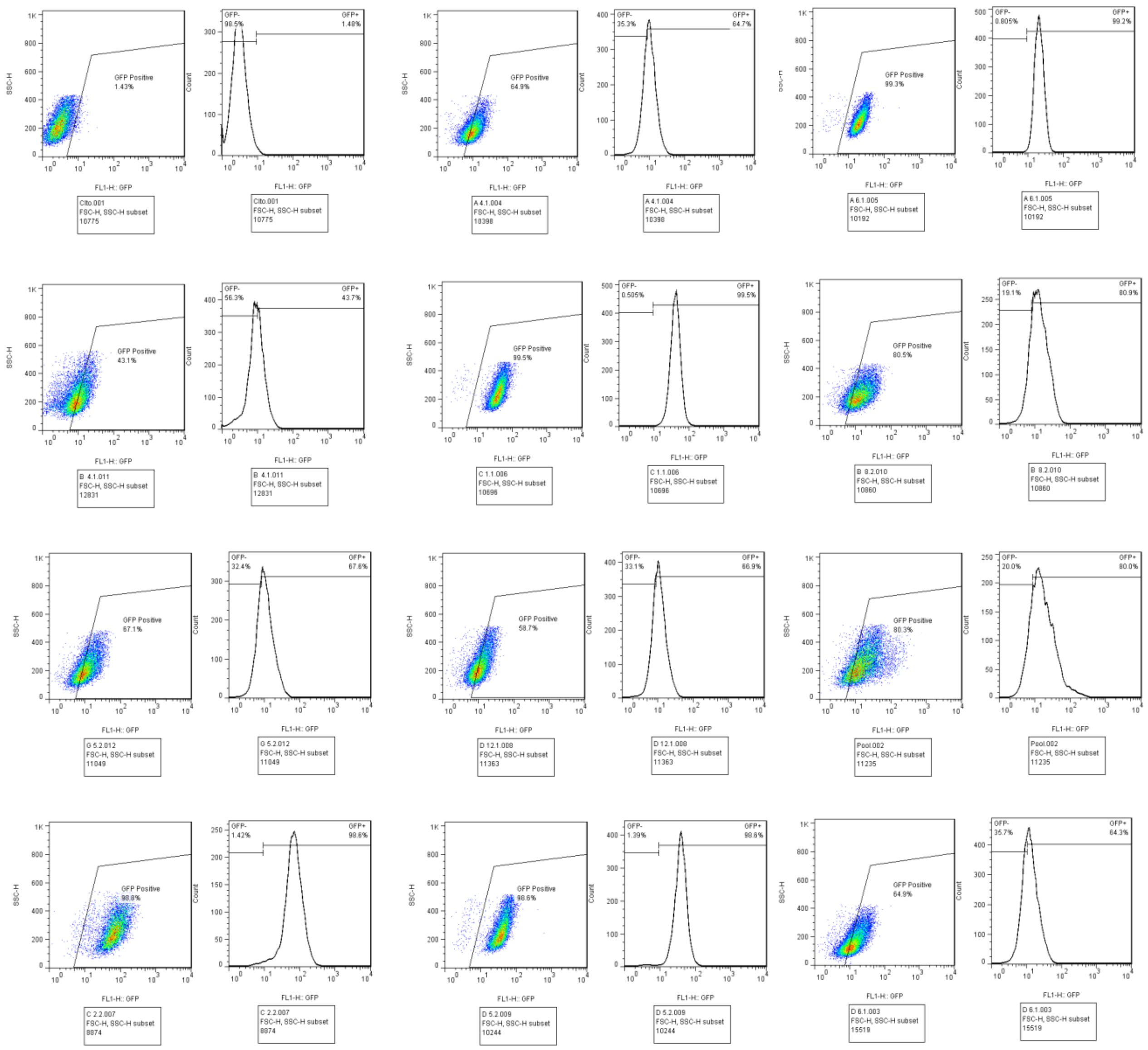

Fig.S1 The flow cytometry results of CHO-Afli single clone screening based on percentage of GFP positive population. Following limiting dilution of cell pool. The single clones detached and analyzed using flow cytometry system.

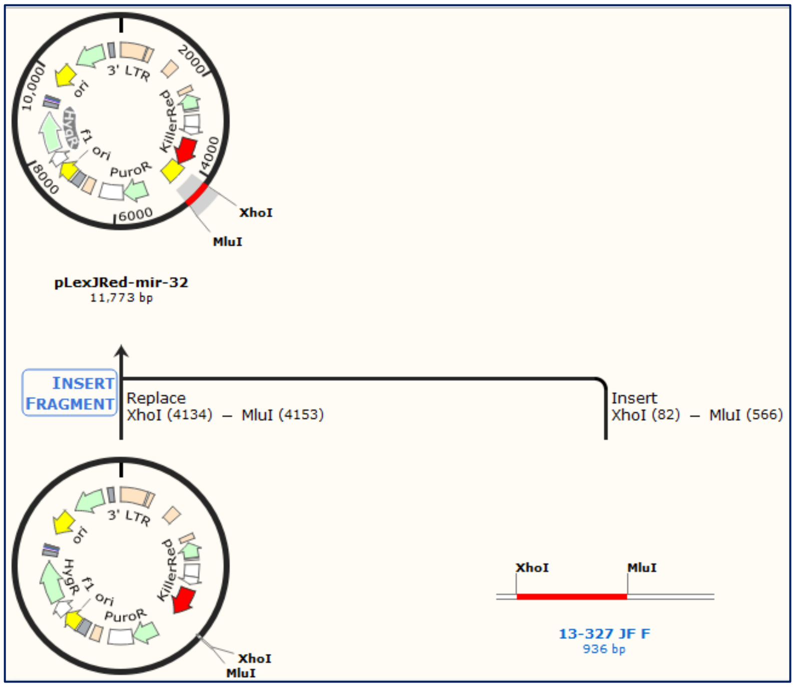

Fig.S2 Schematic picture of constructed mir-32 expression plasmid. The purified PCR product of mir-32 gene was double digested with two different restriction enzymes and was inserted into the pLexJRed vector between the *MluI* and *XhoI* sites.
